# Effects of Piracetam on Pediatric Breath Holding Spells: A Randomized Double Blind Controlled Trial

**Published:** 2012

**Authors:** Ali ABBASKHANIAN, Sara EHTESHAMI, Sadegh SAJJADI, Mohammad Sadegh REZAI

**Affiliations:** 1 Pediatric Neurologist, Assistant Professor, Booali Sina Hospital, Mazandaran University of Medical Sciences, Mazandaran, Iran; 2 General Practitioner, Education Development Center, Mazandaran University of Medical Scineces, Mazandaran, Iran; 3 Pediatrician, Mazandaran University of Medical Sciences, Mazandaran, Iran; 4 Assistant Professor of Pediatric Infectious Disease, Booali Sina Hospital, Mazandaran University of Medical Sciences, Mazandaran, Iran

**Keywords:** Piracetam, Breath-holding spells, Placebo, Treatment

## Abstract

**Objective:**

Breath holding spells (BHS) are common paroxysmal non-epileptic events in the pediatric population which are very stressfull despite their harmless nature. There has been no specific treatment found for the spells yet. The aim of this study was to evaluate the efficacy of piracetam (2-oxo-l-pyrrolidine) on these children.

**Materials & Methods:**

In this randomized double blind clinical trial study, 150 children with severe BHS referred to our pediatric outpatient service were enrolled from August 2011 to July 2012. The patients were randomized into two equal groups.

One received 40mg/kg/day piracetam and the other group received placebo, twice daily. Patients were followed monthly for three months. The number of attacks/month before and after treatment were documented.

**Results:**

Of the enrolled patients, 86 were boys. The mean age of the patients was 17 months (range, 6 to 24 months). In the piracetam group, 1 month after treatment an 81% response to treatment was found. In the placebo group, none of the patients had complete remission and 7% of the cases had partial remission. Overall, control of breath-holding spells was observed in 91% of the patients in the group taking piracetam as compared with 16% in the group taking placebo at the end of the study. There was no significant difference detected between the groups regarding the prevalence of drug side effects.

**Conclusion:**

A significant difference was detected between piracetam and placebo in prevention and controlling BHS. Piracetam (40mg/kg/day) had a good effect on our patients.

## Introduction

Breath Holding Spells (BHS) are common in the pediatric population. They are paroxysmal non-epileptic events affecting 0.1% to 4.6% of otherwise healthy children (1-3). The spells most commonly begin in the first 6 to 12 months of life and almost always by 2 years of age. In 90% of children the spells got remission by school age and the persistence is extremely rare. There are two types of cyanotic and pallid breath holding spells. Some children have both of these spells at some time of their lives (1,3,6).

Typically, BHS begins with a series of stereotypic movements followed by complete or active exhalation which may cause loss of consciousness in the child.

These reactions may be brought on by pain or by strong emotions, such as fear or frustration (2,3). The mechanism of BHS yet remains controversial. The presence of autonomic imbalance with cerebral anoxia, anemia and genetic disorders may be responsible in these spells (2,3,6). No definite therapy has been mentioned for breath holding spells in children, although many studies have investigated different treatments. Reduction in the frequency of spells following treatment with iron supplements in cases of iron deficiency anemia, atropin in pallid spells and piracetam in repeated spells have been reported (4,10,13,15,16). Piracetam (2-oxo-l-pyrrolidine) is a cyclic derivative of gammaaminobutyric acid (GABA) obtained after the loss of one molecule of water followed by ring formation. It has been used for various cognitive disorders and BHS in children (7,20,21). However, conflicting results and few studies on the effect of piracetam in BHS have been published in the literature (8-10,14,). This study was designed to evaluate the efficacy of piracetam compared to placebo in the prevention of severe BHS in children.

## Materials and Methods

This was a randomized double blind clinical trial study on children with severe BHS referred to our pediatric outpatient service in the age range of 6 months to 60 months (5 years) from August 2011 to July 2012.

Diagnosis of severe BHS was based on the history of BHS taken from the parents (three or more spells in 1 month) before entry into the study protocol, defined by the following clinical sequence: provocation followed by crying to a point of noiselessness and accompanying change of color (cyanotic, or pallid) and ultimately a loss of consciousness with an associated alteration in body tone (1,2).

Physical examination was performed in all children and a detail history about their parents was taken. Blood samples were obtained from all patients (CBC, FBS, Ca, Mg and renal function tests) and EEG was done to exclude patients with seizure disorder. All children with identified pallid spells underwent an electrocardiogram to exclude a prolonged QT interval. Children with the diagnosis of epilepsy, electrolyte disturbance, hypoglycemia, iron deficiency anemia, impaired kidney function tests, those with abnormal neurological findings during examination, those who received or were receiving any medications for BHS, or those with a doubtful diagnosis were excluded from the study. The patients were randomized into two groups of 75; those receiving piracetam (40mg/kg/day) and those receiving placebo, which was similar to the piracetam suspension in color and taste, on a randomized basis, twice daily for 3months. Follow-up continued until 3 months after cessation of therapy and no relapse was reported after 3 months of therapy and 3 months of follow-up. 

Both the patients and the researchers were blind to the method of selection and the treatment the groups were receiving (double-blinded). The frequency of attacks was recorded according to the information given by the parents.

The patients attended the clinic at the beginning of the study and were then followed monthly for a total period of 3 months. The final assessment was carried after 3 months after inclusion in the study. Typical symptoms of BHS were described to the parents of the patients. The frequency of attacks was based on the number of episodes during that period that had been reported by the mother or any other family member taking care of the child.

At the end of the three months, the response to treatment was evaluated. We defined response as follows: 

1-“Complete response”, the attacks disappeared completely, 2-“Partial response; more than 50% reduction in the attacks and 3-“No response”; no or less than 50% reduction in the attacks.

The study was registered by the IRCT with IRCT ID: IRCT138801231808N1 and was approved by the ethical committee of Boo Ali Sina Referral Pediatric Hospital. Informed consent was obtained from all the parents. Data were collected from the patients in the form of a questionnaire which consisted of demographic data of the children and some questions including the frequency of attacks/month, the type of spells and side effects of the drugs. Student’s t-test and Chi-square test were used for statistical evaluation. Wilcoxon signed rank test was used to compare the number of attacks before and after treatment in each of the studied groups; while Mann-Whitney test was used to compare the response of treatment between piracetam and the placebo groups. The data were analyzed using proper statistical test with SPSS 16.0 for Windows. The significance level was set at p<0.05. 

## Results

One-hundred seventy-eight patients were enrolled into the study and a total of 28 patients were lost to follow-up after treatment and 150 (86 boys, 64 girls) completed the study. The ratio of boys to girls was 1.3: 1. Most of the patients (84%) in the piracetam group and (80%) in the placebo group were aged between 6 and 24 months. 

The patients were divided into two groups (A and B). At the end of the study, it was found that group A were patients receiving piracetam and group B was the placebo group. The characteristics of the treatment groups are delineated in Table 1. 

Frequency of spells varied widely, ranging from 15 per day to two per month and the mean frequency was three episodes of severe BHS in one week. The duration of each spell was difficult to assess with accuracy. Most of them were reported to last less than 2 minutes by the parents. The number of spells before treatment was not different between the groups (p>0.05).

There was no significant difference between the types of BHS (cyanotic, pallid and mixed) between the two groups. In 110 cases, the spells were cyanotic type; in 30 cases, pallid; and in 10 children they were mixed type.

The provocation factor was not significantly different in the two groups (p=0.52); in 91 children it was crying, in 34 it was pain, in 15 it was anger and in 10 patients it was head bump.

Consanguineous marriages and family history did not differ between the two groups (p=0.084). 

In 26 children, the parents had a familial relationship (nephew and other relationships); 14 children in group A and 12 children in group B. A positive family history was detected in 12 children in group A and 16 children in group B, but no statistically significant difference was detected regarding this matter between the two groups (p=0.31).

EEGs were all normal except for 11 patients in which slight paroxysmal activity or dysrythmias were detected.

One month after treatment with piracetam, response to treatment was 81% in the piracetam group (47% complete response, 34% partial response) and in 19% there was no response. In the placebo group, none of the patients had complete response, 7% had partial response and 93% no response. Sixty-four percent of the piracetam group and 4% of the placebo group showed complete response two months after treatment. Partial response was seen in 25% of the piracetam group and 9% of the placebo group. Eleven percent of the piracetam group and 87% of the placebo group had no response.

Three months after treatment, complete response was seen in 77% of the piracetam group and 6% of the placebo group; partial response was seen in 14% and 10%; and no response was seen in 9% and 84% of the patients, respectively. Response to treatment was assessed after 3 months in both groups and the results are shown in Table 2.

**Fig. 1:**
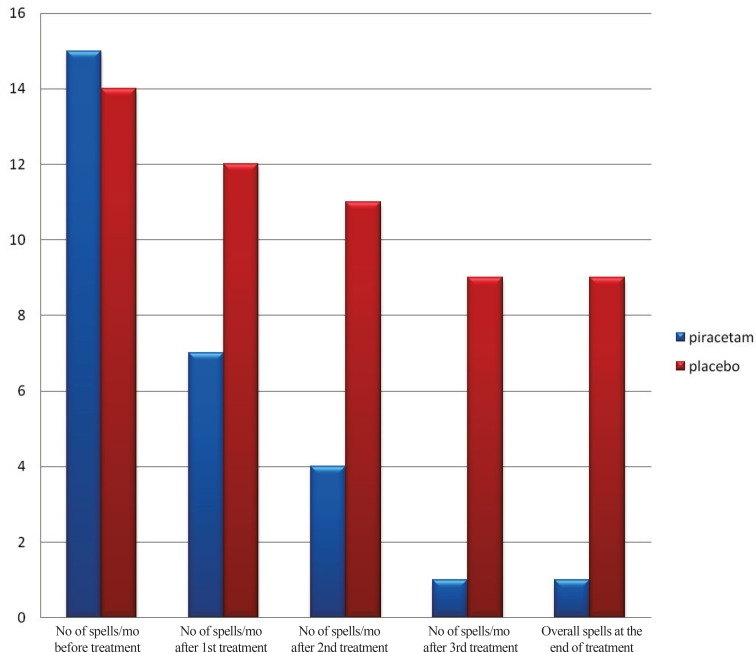
Number of attacks/month throughout the study period in the two groups (median values)

There was no significant difference between types of BHS (cyanotic and pallid) and the rate of change after treatment in the two groups. The number of attacks/ month, the overall number of attacks/month after treatment, rate of change after the first month and the whole rate of change for the two groups are shown in Figure 1.

The prevalence of drug side effects did not show any significant difference between the two groups. In children who received piracetam, 2 cases of vomiting and 2 cases of emotional liability were seen and in the placebo group there were just 2 cases of vomiting and no other side effect was detected in the control group. 

## Discussion

BHS has been reported to occur in approximately 0.1% to 4.6% of well children. Most pediatricians agree to find out an effective drug despite reassurance for prevention of these spells (1,3,4). in this way some treatments from Chinese herbal medicine to cardiac pacing, antiepileptic and atropine with variable results have been advised. Some studies have reported the use of piracetam in children with BHS (10-13,15). 

The age of onset of BHS in most of the studies has been in the first 12 months of life. The occurrence of breath-holding spells is rare in the first 6 months of life and questionable in the neonatal period (1,5,6,). In our study, in 78% of the cases, the spells developed at the age of 6 to 24 months and the onset of spells in 58% of the cases was within the first 12 months of life, which is consistent with the above mentioned studies. 

The ratio of boys to girls was 1.3:1 which was similar to studies conducted by DiMario, Donma and Ashrafi et al. (1,10,14). 

BHS is provoked by frustration, anger, fear or pain. In our study, crying and pain were the common triggering factors (84%) and in both groups, cyanotic spells were the most common type. These results were in agreement with previous reports (1,3,6).

A 20-30% rate of positive familial history in children with breath holding spells indicates that genetic may be the causality factor for occurrence of these spells (1,6,19). In our study, in 28% of the children, the parents had a familial relationship and in 18% of the children, the parents had a positive familial history of BHS.

The frequency of BHS varies from multiple episodes per day to as infrequently as on a monthly basis. The majority of children; however, experience multiple episodes per week. In the study of Di Mario (1) 24/95 (25%) of the patients experienced more than 1 episode per day during peak frequency. In an Indian study performed by Bhat et al. (5), only 18% of the patients had more than one episode of BHS per day and 64% of the patients had multiple episodes occurring every week at the time of peak frequency. In our study, only 24% of the patients had more than one episode of BHS per day and 68% of the patients had multiple episodes occurring every week at the time of peak frequency. This discrepancy may have been caused by iron deficiency anemia among patients in the other studies. In our study, we found significant improvement after administration of piracetam, but not after placebo. The significant decline in the number of attacks/month after administration of piracetam was marked in the second and third months, and it was less pronounced in the first months when compared with the second and third months. The overall control of BHS was observed in 91% of the patients (complete and partial response) in the group taking piracetam as compared with 16% in the group taking placebo (p<0.05) (Table 2). 

Garg (9) has also shown that 2 months piracetam therapy reduced the spells significantly and concluded that the drug is safe and effective. In the study carried out by Aazam et al. (8), the efficacy of piracetam was identical (90%) with a relatively higher dose (50-100 mg/kg/day), but these authors did not have a control group for their study. Similar results were obtained in the study of Donma et al. (10), in which the spells were controlled in 92.3% of the patients treated with piracetam and 29.7% of the placebo group patients. In a study conducted by Ashrafi et al. (14), piracetam in comparison with placebo did not show particular advantage. The authors considered the periodic nature of these attacks and they emphasized that a response or no response in a limited course is not a reliable criterion for accepting or rejecting a drug. 

Two large scale multicenter studies in children showed no serious adverse effects of piracetam; however, emotional lability and allergic dermatitis were reported occasionally (17,18). In the present study, no serious side effects were reported by the parents; few, however, vomiting and emotional lability were seen in the piracetam and placebo group. No allergic reaction, hematological, biochemical, or liver function abnormalities were seen. 

According to the mechanism of spells, in which the extended tissue anoxia will lead to reduction of the patients consciousness, it seems piracetam with its effect on increasing tissue oxygenation and increasing the inhibitory process of tissue hyperpolarization, similar to GABA, may be helpful in controlling spells (2,3,20,21).

Some studies showed that attitude problems in the mother and the child may trigger spells and with proper psychotherapy consultation with the parents, especially mothers, these spells may be prevented to a large extent (4).

**Table 1:** Patients Characteristics

	Group A (Piracetam) (n==75)	Group B (Placebo) (n==75)
Mean age at onset (months)	10.9	10.5
Gender		
Male	40	46
Female Consanguinity Positive Family History	33 19 12	31 23 16
Type of Spells		
Cyanotic	82%	78%
Pallid	8%	16%
Mixed	10%	6%

**Table 2:** Response to Treatment in Groups

** Response**	**After 1 Month**			**After 2 Months**			**After3 Months**		
**Group**	Complete response	Partial response	No response	Complete response	Partial response	No response	Complete response	Partial response	No response
**Piracetam**	47%	34%	19%	64%	25%	11%	77%	14%	9%
**Placebo**	0%	7%	93%	4%	9%	87%	6%	10%	84%

**In conclusion,** for simple BHS, parental reassurance would suffice; however, children with severe and repeated BHS should be given piracetam prophylaxis and piracetam (40mg/kg/day) is a safe and effective drug for the treatment of breath-holding spells in children. Although our center was a referral center and we finally had about 150 patients in our study, we recommend this study with extended randomized placebo-control trials and more duration follow-up, optimal dose and length of treatment should be considered to evaluate longterm benefits and serious side effects of piracetam in the prevention of severe BHS.

## References

[1] DiMario FJ Jr (2001). Prospective study of children with cyanotic and pallid breath-holding spells. Pediatrics..

[2] Kotagal P, Costa M, Wyllie E, Wolgamuth B (2002). Paroxysmal non epileptic events in children and adolescents.

[3] Kolkiran A, Tutar E, Atalay S, Deda G, Cin S (2005). Autonomic nervous system functions in children with breathholding spells and effects of iron deficiency. Acta Pediatric..

[4] Hüdaoglu O, Dirik E, Yiş U (2006). Parental attitude of mothers, iron deficiency anemia, and breath-holding spells. Pediatr Neurol..

[5] Ahmad Bhat M, Ali W, Mohidin K, Sultana M (2007). Prospective study of severe breath holding spells and role of iron. J Pediatr Neurol..

[6] Lombroso CT, Lerman P (1967). Breath holding spells (cyanotic and pallid infantile syncope). Pediatrics..

[7] Gouliaev AH, Senning A (1994). Piracetam and other structurally related nootropics. Brain Res Rev..

[8] Azam M, Bhatti N, Shahab N (2008). Piracetam in severe breath holding spells. Int J Pschyiatry Med..

[9] Garg RK Piracetam for the treatment of breath holding spells. Indian Pediatrics.1998 Oct.

[10] Donma MM (1998). Clinical efficacy of piracetam in treatment of breath holding spells. Pediatr Neurol..

[11] Murata R, Matsuoka O, Hattori H, Kawawaki H, Nakajima S, Nakamura M et al (1988). Efficacy of Kan-bakutaiso-to (TJ-72) on breath-holding spells in children. Am J Chin Med..

[12] Kelly AM, Porter CJ, Mc Goon MD, Espinosa RE, Osborn MJ, Hayes DL (2001). Breath-holding spells associated with significant bradycardia: successful treatment with permanent pacemaker implantation. Pediatrics..

[13] McWilliam RC, Stephenson JB (1984). Atropine treatment of reflex anoxic seizures. Arch Dis Child..

[14] Ashrafi MR, Mohammadi M, Shervin Badve R (2002). Efficacy of piracetam in treatment of breath-holding spells Iran. J Pediatr..

[15] Daoud AS, Batieha A, al-Sheyyab M, Abuekteish F, Hijazi S (1997). Effectiveness of iron therapy on breath-holding spells. J Pediatr..

[16] Ziaullah Nawaz S, Shah S, Talaat A (2005). Iron deficiency anemia as a cause of breath holding spells. J Postgrad Med Instit..

[17] Di lanni M, Wilsher CR, Blank MS, Conners CK, Chase CH, Funkenstein HH et al (1985). The effects of piracetam in children with dyslexia. J Clin Psychopharmacol..

[18] Wilsher CR, Bennett D, Chase CH, Conners CK, Dilanni M, Feagans L et al (1987). Piracetam and dyslexia: effects on reading tests. J Clin Psychopharmacol..

[19] DiMario FJ Jr, Sarfarazi M (1997). Family pedigree analysis of children with severe breath-holding spells. J Pediatr..

[20] Winnicka K, Tomasiak M, Bielawska A (2005). Piracetam-an old drug with novel properties. Acta Pol Pharm..

[21] Winblad, B Piracetam: a review of pharmacological properties and clinical uses. CNS drug rev..

